# Contrasting Responses of Alien and Ancient Forest Indicator Plant Species to Fragmentation Process in the Temperate Lowland Forests

**DOI:** 10.3390/plants11233392

**Published:** 2022-12-06

**Authors:** Mirjana Šipek, Lado Kutnar, Aleksander Marinšek, Nina Šajna

**Affiliations:** 1 Faculty of Natural Sciences and Mathematics, University of Maribor, 2000 Maribor, Slovenia; 2 Slovenian Forestry Institute, 1000 Ljubljana, Slovenia

**Keywords:** forest fragment, indicator plants, invasion, diversity, habitat modification, habitat conservation

## Abstract

Fragmentation is one of the major threats to biodiversity. In a fragmented landscape, forest specialists are losing suitable forest habitats with specific site and microclimate conditions, which results in their local extinction. Conversely, the invasion of alien species is facilitated by open forest areas and increased boundaries between forest fragments and adjacent land. We studied the effect of fragmentation in terms of fragment size impact on overall plant species richness and on selected ecologically important groups’ richness, composition, and diversity. We surveyed vegetation in the interior of 47 fragments of various sizes and one unfragmented reference forest. Our results reveal that the effect of fragmentation is complex and differs for studied plant groups. Decreasing fragment size negatively affects the overall plant richness and richness of native and ancient forest indicator plants as well as their diversity, while the effect is positive for alien plants. The highest proportion of ancient forest indicator plant species and the lowest proportion of alien plants in the unfragmented forest underline the great conservation value of forest fragments. At the same time, our results reveal that large and diverse forest ecosystems are susceptible to biological invasions as well.

## 1. Introduction

Fragmentation has a complex effect on biological communities by altering the biotic and abiotic components of an ecosystem [1]. The most evident negative effects of forest fragmentation are habitat loss, wide forest edge formation, declining connectivity, and consequent isolation of populations [2,3]. The effect of fragmentation on biota is either negative, neutral, or positive [4,5]. The abundance of certain species may decline sharply, or they may even become locally extinct, and other species may remain stable, while the abundance of some may increase dramatically [3].

To understand the effect of habitat fragmentation on species richness, island biogeography theory provides a theoretical framework [6,7]. A fragmented landscape is considered a dynamic system where species are disappearing and appearing on a local scale (fragment level) because of extinction and immigration processes, respectively. The intensity of each process depends on fragment size, isolation, and connectivity. Larger fragments tend to have higher species richness, species extinction is slower, and they do not suffer as much from the negative consequences of isolation (e.g., lost connectivity within a metapopulation) in comparison to smaller fragments [6].

Considering the island biogeography theory, some forest species are expected to disappear in smaller forest fragments and an influx of species from the surrounding landscape, which is a source of colonizers, is expected [8,9]. Forest specialist species in small and isolated forest fragments are subjected to decreased fitness because of a lack of pollinators [10] or inbreeding following population isolation [11,12,13]. This may result in lower seed set and abundance [13] and eventually cause local extinction of these species. Ancient forest indicator plant species (AFI) is a group of forest specialist plants that differ ecologically from other forest plants. They are poor colonizers, characterized by greater shade and stress tolerance with predominant geophyte and hemicryptophyte life-form [14]. Therefore, Hermy et al. [14] suggested that the presence of AFI may serve as an important biodiversity species indicator for forests.

Moreover, the fragmented landscape is highly susceptible to biological invasions because of the increased boundary between forest and adjacent non-forest land [15,16,17]. Therefore, the entry of non-forest and alien species into forest fragments is among the most commonly reported observations [2,3,18,19]. In smaller and more isolated forest fragments, species turnover should appear to a greater extent than in larger, more connected fragments [3,17]. This process is especially important in light of nature conservation because, in an anthropogenically modified landscape, fragmentation and biological invasions are one of the main causes of forest ecosystem degradation [20,21]. However, the effect of fragment size on plant species richness is highly variable, showing a lack of consistency [1,9,22,23].

Slovenia is among European countries with the highest share of forests, comprising around 60% of the country’s area [24,25]. Larger, continuous forested areas prevail in hilly areas, while lowland forests are more fragmented, consisting of various spatial configurations with the most prominent difference among them—the fragment size. Forest fragments embedded in the agricultural matrix and intertwined with urbanized areas host alien plant species, among them several invasive ones [19]. Lowlands in NE Slovenia are an appropriate landscape to study fragmentation effects because forest fragments of various sizes are placed in a relatively homogenous area with similar abiotic conditions regarding soil and climate. Current research has shown that the invasion level of lowland forests differs among various forest habitat types [26], while the effect of the forest fragments’ size on invasion level is yet to be studied. Moreover, Marinšek and Kutnar [26] reported that invasive alien plants have a negative effect on the regeneration of native forest species. Therefore, a better understanding of the spatial characteristics of forest fragments in the invasion process is needed for appropriate management planning to prevent new invasions and to enable the conservation of forest ecosystems.

In our study, we evaluated the fragmentation process with two descriptors: i) forest fragment size and ii) the proportion of forest in a buffer zone of 500 m in the surroundings of the forest fragment as a surrogate for isolation/connectivity. The effect of fragment size on plant richness per unit area is less predictable [15] than per the whole fragment, where the correlation is consistently positive because of increasing habitat heterogeneity [27]. We expect that the effect of forest fragment size among different ecological plant groups varies. The study aimed to test whether the size of the lowland forest fragment affects (1) plant species richness of overall vascular plants, with special consideration to native, alien, alien invasive, and ancient forest indicator species, (2) diversity per unit area, and (3) plant species composition expressed as a ratio between different ecological groups.

## 2. Results

### 2.1. Floristic Richness and Composition

In the forest fragments at Apaško polje and Dravsko polje, we found 124 native plant species (37 AFI) and 17 alien plants, 10 of which are considered invasive (Table 1).

In the unfragmented forest, 87 native (30 AFI) and 9 alien plants were listed. Among aliens, 6 were invasive, while the remaining 3 alien tree species (*Juglans nigra, Fraxinus americana* and *Populus x canadensis*) were planted in the Murska šuma forest for forestry purposes in the past and, in Slovenia, are considered as naturalized (Table 1).

Of the overall identified alien plants (21), 5 were common in unfragmented forest and smaller fragments across Dravsko and Apaško polje. Those are *Acer negundo*, *Erigeron annuus*, *Impatiens glandulifera*, *I. parviflora*, and *Solidago gigantea*, all of them invasive.

### 2.2. Effect of Fragment Size on Plant Species Richness

**Figure 1:**
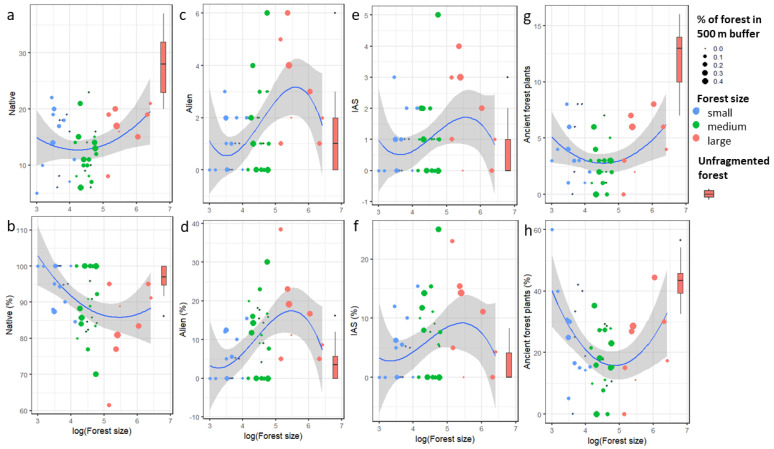
Effect of forest fragment size on richness (**a**) and proportion (**b**) of native plant species, alien (**c**,**d**), invasive alien (**e**,**f**), and ancient forest plants (**g**,**h**) in a plot. The color and size of the dots correspond to the forest fragment size group and % of forest cover in 500 m buffer, respectively. Control unfragmented forest Murska šuma is shown by a bar (minimum, 1st quartile, median, 3rd quartile, maximum).

**Figure 2:**
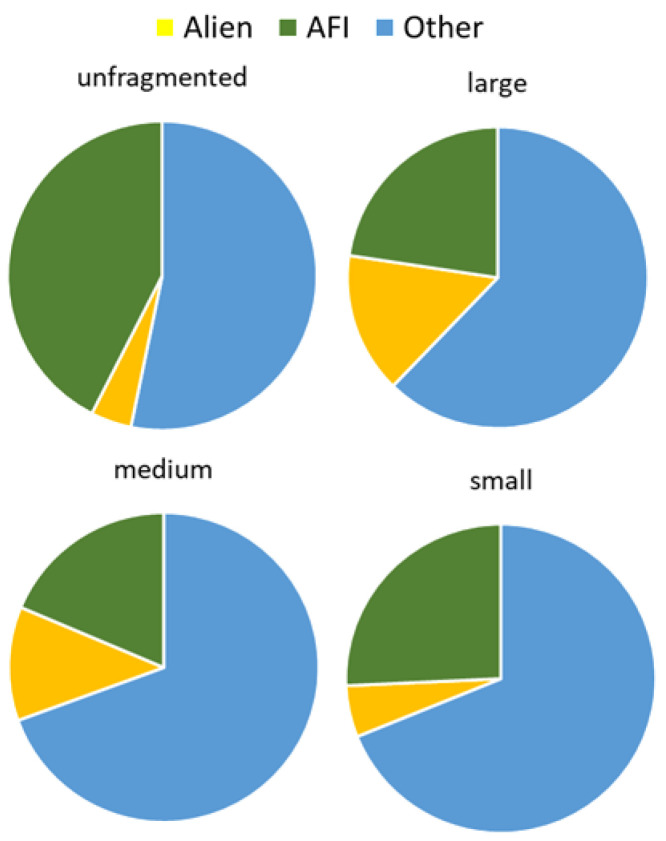
Plant composition quality assessed by an alien, AFI, and other plants in forest groups according to their size: unfragmented forest, large, medium, and small forest fragments.

We found a significant effect of fragment size on the richness and percentage of alien plants, percentage of native, and percentage of AFI (Table 2, Figure 1). Small fragments had a high number of AFI (Figure 2), which interfered with the models for native plants and their subgroup AFI (Figure 1a,b,g,h). With the increasing size of large fragments, the richness, proportion, and cover of alien plants start to fall toward the value of the unfragmented forest. This is also true for IAS (Table 2 and Table 3, Figure 1).

### 2.3. Effect of Forest Fragment Size on Plant Species Composition Quality

**Figure 3:**
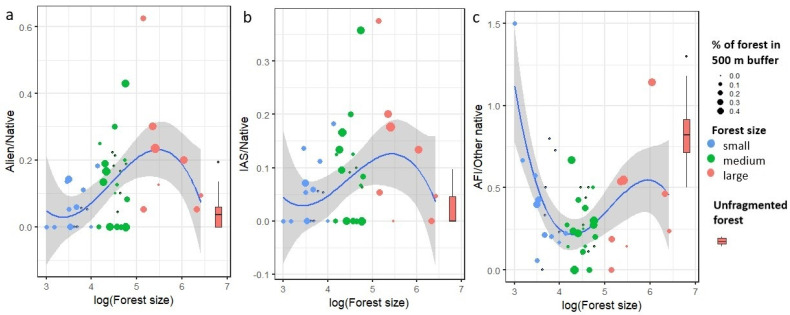
Effect of forest fragment size on plant composition assessed by the ratio between (**a**) alien and native plants, (**b**) invasive alien and native plants, and (**c**) ancient forest indicator plants and the rest of native plants. The color and size of the dots correspond to the forest fragment size group and % of forest cover in 500 m buffer. Control unfragmented forest Murska šuma is shown by a bar (minimum, 1st quartile, median, 3rd quartile, maximum).

The ratio of alien and native as well as IAS and native plant species increases with increasing forest size. However, for large forest fragments larger than 30 ha, the curve bands toward the values for the unfragmented forest. The opposite trend was found for the ratio of AFI and other native plants. According to regression analysis, the significance was shown only for the composition of alien/native and AFI/other native plants (Table 2, Figure 2 and Figure 3).

Considering plot level, significantly higher richness, proportion, and cover of AFI was found in the unfragmented forest. In the same forest, the lowest proportion and cover of alien plants and IAS was detected (Figure 1, Table 3).

### 2.4. Effect of Forest Fragment Size on Diversity

**Figure 4:**
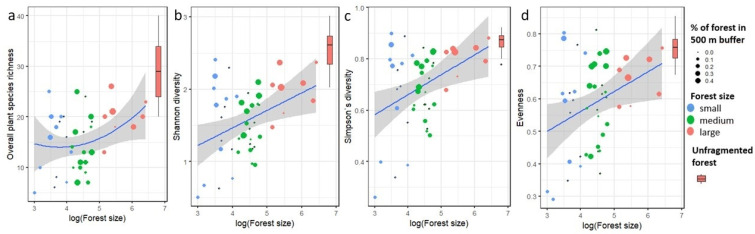
Effect of forest fragment size on plant diversity evaluated by (**a**) plant richness, (**b**) Shannon diversity, (**c**) Simpson’s diversity, and (**d**) evenness. The color and size of the dots correspond to the forest fragment size group and % of forest cover in 500 m buffer. Control unfragmented forest Murska šuma is shown by a bar (minimum, 1st quartile, median, 3rd quartile, maximum).

We found a significant positive effect of forest fragment size on overall plant species richness and plant diversity assessed by Shannon diversity, Simpson’s diversity, and evenness (Table 2 and Table 4, Figure 4).

Species richness increased by 32–54%, Shannon diversity increased by 22–32%, Simpson’s index by 8–25%, and evenness by 12–28% if we compare different-sized forest fragments to the unfragmented forest (Table 4).

## 3. Discussion

Fragmentation of the European forests is ongoing nowadays. After 2015, an abrupt increase in forest harvest was recorded in the majority of European countries [25], including Slovenia [29]. Given this trend, it is even more important to understand better the impact of fragmentation and reduction of forest fragment size on its biodiversity. In this study, we focused on responses of alien and ancient forest indicator plant species according to forest fragment size, because these two plant groups may well indicate ecosystem degradation and preservation, respectively. Moreover, the study area is also the habitat of several endangered plant species such as *Gagea spathacea* (Hayne) Salisb., *Carex acutiformis* Ehrh., *C. riparia* Curt., and *Chimaphila umbellata* (L.) W. Barton (our unpublished records). This further supports the effort to study changes in species composition in such a landscape.

If we compare the total sampled area of all forest fragments to the total sampled area of a large unfragmented forest, higher species richness confirmed high cumulative species number for the fragmented sampled area. A similar observation was found by Herrera and Laterra [30] when assessing the effects of fragmentation on grasslands. This biodiversity and biogeography phenomenon is well known as distance decay similarity [30]. In a larger spatial extent, differences in species composition increase due to larger environmental heterogeneity and dispersal limitation [27,31].

Our results confirm that forest fragment size affects plant community composition besides other taxa such as beetles [32], birds [33,34], small mammals [35], and others. Larger forest fragments contain higher richness and a higher proportion of AFI per unit area, compared to smaller forest fragments, while there are fewer alien plants. Higher plant diversity in larger forest fragments and plant community composition compared to smaller fragments support the importance of forest fragment size in a fragmented landscape.

The pan-European survey revealed the vulnerability of forest habitats to alien plant invasions and predicted the further spread of alien species, especially under certain circumstances such as fragmentation [36]. The invasion of alien species affects biodiversity by reducing the richness and abundance of native species and alters several ecosystem functions [37]. Invasive alien plants overcome biogeographic barriers and cause plant communities’ homogenization [38]. Moreover, they can form dense stands in forest gaps and aggravate the natural rejuvenation of the native forest tree species [26]. Therefore, it is critical to understand how forest fragment size, as the most obvious change in fragmented landscapes, affects alien plant species, including invasive species. We would expect smaller forest fragments to be more susceptible to alien plant invasion because the forest edge has proportionally more contact with the surrounding non-forest matrix, which is a donor for alien plants. However, our results show that in the case of small forest fragments, species richness was either overestimated or underestimated. If extinction rates are slow and species from the matrix penetrate forest fragments, species richness can be in smaller forest fragments for at least temporary enhancement [1]. For example, species richness of native plant species in small forest fragments in our study was inflated because of high light availability, allowing the establishment of native non-forest specialist plants. Additionally, we have shown a high richness of AFI species in small forest fragments because AFI species are long-lived plants. On the other hand, these temporary higher richness values for native plants give the impression that in small forest fragments, alien species have low abundance and richness, particularly if we compare the native vs. alien species ratio.

Our results confirm the general presence of different alien plant species in the lowland forest ecosystems regardless of their size. Forest fragment size did affect alien, but not invasive alien plant, richness. The highest richness, proportion, and a cover of aliens the invasive plants among them were recorded in larger forest fragments, while their presence was the lowest in unfragmented and small fragments. The level of invasion does not solely depend on forest fragment characteristics but is a subset of many variables including matrix quality [3,16], which is the most likely reason for such a result in our study. The most common alien plant in sampled forest fragments was *Solidago gigantea*, which is recognized as one of the invasive plants with the highest impact in Europe [39]. It was present in 26% of plots in fragmented and in 34% of the unfragmented forest.

Ancient forest indicator plant richness, relative abundance, and cover significantly increase with increasing forest fragment size. According to our dataset, the AFIs have confirmed their indicative value, but only for fragments larger than 6 ha due to the long lifetime of the AFIs. Unfragmented forest, serving as a control site, had the highest share of native and ancient forest indicator plant species in comparison to smaller forest fragments of various sizes. Our results indicate species turnover in sampled forest fragments. Even if a proportion of native species was similar in all plots, their composition differed at the expense of AFI, whereby once more the indicative value of this plant group is evident. This is consistent with findings from the mixed-oak forest in the northern Iberian Peninsula, where fragmentation mainly negatively affected the diversity of herbaceous forest specialist species, while other growth forms were less affected [40]. The study of Naaf et al. [13] provides a great insight into the population biology of three ancient forest indicator plants as a response to habitat fragmentation, all of them also present at our study site: *Oxalis acetosella*, *Anemone nemorosa* L., and *Polygonatum multiflorum*. All three herbs showed sensitivity to habitat fragmentation and isolation, which was species-specific due to different life-history traits. Habitat fragmentation resulted in a smaller population size and lower genetic diversity of these herbs. However, because of vegetative reproduction and long lifespan, the negative effects of habitat loss for some understory plants may be observed decades after forest fragmentation [41]. We can expect this to be true in our study for *Adoxa moschatellina* L., *Anemone nemorosa,* and *Paris quadrifolia*, as all three herbs also persisted in smaller forest fragments.

The high cumulative numbers of native plants, including AFI, illustrate the conservation value of small forest fragments in a predominantly biodiversity-depleted agricultural matrix. Our results have shown that they must have a critical size to maintain their species conservation area in the future. In our study area, the critical size was 13 ha. Unfragmented forest serving as a control site had the highest share of native and ancient forest indicator plant species in comparison to smaller forest fragments of various sizes. Moreover, in the unfragmented forest, although not entirely without alien plants, their overall richness, proportion, and cover per plot were lower than in smaller forest fragments. To summarize these findings, we must attribute higher conservation value to the unfragmented forest and maintain forest fragments of at least 13 ha in the studied agricultural landscape.

## 4. Materials and Methods

### 4.1. Study Area

**Figure 5:**
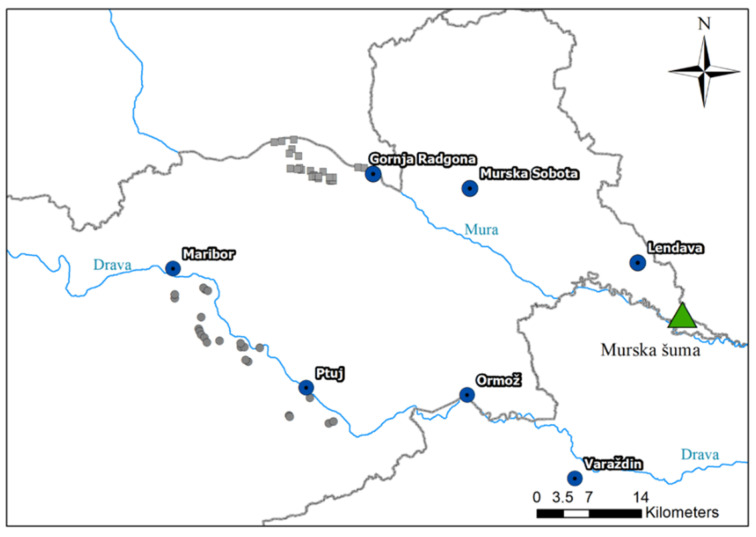
Map indicating the study area in the NE part of Slovenia, Europe. In detail, the distribution of forest fragments in studied areas is indicated: Apaško polje (grey squares) and Dravsko polje (grey dots). The control of the unfragmented forest area of Murska šuma is indicated by the green triangle.

The study area consists of forest fragments of various sizes (Illyrian *Quercus-Carpinus betulus* forest by EUNIS habitat classification) [42] located on the planes of Apaško polje and Dravsko polje near rivers Mura and Drava, respectively, in NE Slovenia (Figure 5). Apaško polje has an average elevation of 220 m a.s.l., while the average elevation of Dravsko polje is 250 m a.s.l. Both plains are part of the Danube River basin in the sub-Pannonian biogeographical region with a temperate continental climate. The 10-year average precipitation is 837 mm and 938 mm at Apaško polje and Dravsko polje, respectively, and the average temperature in both plains is 11.2 °C in the period from 2011 to 2021 [43]. Most of the study area is covered with shallow and sandy soils on deposited gravel or sand, suitable for agriculture, but is sensitive to summer droughts. Prevailing agricultural land use at both plains intertwines with forest fragments and settlements. Apaško polje area has a rural character, while the Dravsko polje area is more urbanized, with larger towns and long roadside settlements [44,45].

Our study also included the extensive forest “Murska šuma” located near the Mura River in the NE Slovenia (Figure 5). The elevation of the area is around 190 m a.s.l., the 10-year average precipitation is 812 mm, and the average temperature is 11.7 °C in the period from 2011 to 2021 [30]. More details about Murska šuma are in Marinšek and Kutnar [26].

### 4.2. Data Collection

According to the satellite imagery (Google Earth Pro) [46] and later field inspection, we selected 23 and 24 forest fragments at Apaško polje and Dravsko polje, respectively. Forest fragments are defined as smaller areas (in this study between 0.1 and 260 ha) with typical forest vegetation surrounded along the entire perimeter with other land use types (e.g., arable land, settlements, roads). We drew polygons of sampled forest fragments using Google Earth Pro to determine the perimeter (forest edge), area, and centroid of each fragment. In Arc GIS [47] we overlaid polygons of forest fragments with layer Corine Land Cover [48] and extracted the area of the forest cover in a 500 m buffer zone surrounding fragments of interest.

We evaluated fragmentation with two descriptors for each studied forest fragment: forest fragment area and connectivity in terms of forest cover in the 500 m buffer zone surrounding each fragment.

Approximately in the centroid of each forest fragment, we surveyed vegetation according to the Standard European method [49] in the summer of 2019. Cover estimation was assessed using the Braun-Blanquet cover-abundance scale. All plots were of 100 m^2^ (10 m × 10 m) to ensure comparability. The size of the plots was determined to avoid the edge and to ensure that even in the smallest fragments forest interior was sampled.

Forest fragments were classified according to their size: large (>13 ha; 8 plots), medium (1.5–13 ha; 23 plots), and small (0.1–1.5 ha; 16 plots) fragments.

To increase the reliability of the study of fragment size effect, we included 29 plots from the extensive forest area of Murska šuma (<900 ha) which was treated as a control site in this study—an unfragmented forest area to serve as “negative control”. Plots were of 200 m^2^ with vegetation belonging to the Illyrian oak-hornbeam habitat type (for more details see Marinšek and Kutnar [26]). Because differences in plot size may affect species richness, we compared species richness as well as the proportions of predetermined plant groups (Table 5). To avoid the influence of the plot size, all comparisons were repeated by weighting the number of plant species by the plot size logarithm (2.3 for 200 m^2^ plots and 2 for 100 m^2^ plots). All trends for the plant species richness remained the same, even after the weight of the plot size. However, we must emphasize that the samples from forest Mura šuma were not included in the regression analysis but served only as a control of the reliability of the direction of obtained trends.

A total area of 4700 m^2^ (representing 5.99% of fragmented forest area) and 5800 m^2^ (representing 6.44% of unfragmented forest area) were sampled in fragmented forests at Apaško polje and Dravsko polje and the unfragmented forest area of Murska šuma, respectively.

### 4.3. Plant Species Classification

We classified plants according to their origin, invasiveness, and fidelity to (old) forest habitats. The origin of the plants was based on Martinčič et al. [50]. We considered only neophytes (plants that were introduced in Europe after 1500) as alien plant species. Plant species that do not naturally occur in the study area but are native in other parts of Slovenia were also considered alien (e.g., *Taxus baccata* which is frequently planted in urban hedgerows). The invasiveness status of alien plants followed expert judgment, according to which plants with naturalization status 5 were considered invasive species [50]. As forest specialist species, we considered slow-colonizing species unable to colonize new forests quickly. Those plants are recognized in Central Europe for having an indication potential and exhibiting high fidelity to old forest stands (ancient forest indicator plants—AFI) [14]. Plant species nomenclature was followed by Martinčič et al. [50].

### 4.4. Data Analysis

Different vegetation layers in the forest were merged into a single one, considering the independence assumption in JUICE software [51]. Plant diversity per plot was quantified by calculating species richness, Shannon index, Simpson index, and evenness in JUICE software.

Descriptive statistics were used to summarize species richness, proportion, and cover of different functional groups of plants per plot in the reference forest and three groups of fragmented forests. Differences among groups were assessed by ANOVA and post-hoc Tukey HSD. If assumptions for parametric tests were violated, we used Kruskal-Wallis and post-hoc Mann–Whitney tests.

Fragment size and % cover of forest in 500 m buffer were significantly correlated (linear regression, *p* < 0.0001). Therefore, only fragment size was included in the models as an explanatory variable.

We built linear (y = a *q + r), quadratic (y = a *q + b *q ^2^ + r), and polynomial (y = a *q + b *q ^2^ + c *q ^3^ + r) regression models (*lm()*) to evaluate the effect of fragment size on plant species richness (overall plant richness, native, alien, IAS, and AFI), composition (alien/native, IAS/native and AFI/other natives), and diversity (Shannon, Simpson’s, and evenness). The final models were selected according to the criterion of the best fit. The distribution of response variables was visualized using functions *ggdensity()* and *hist()*. To test the normality of model residuals, we used a function *plot()*. The level of the significance was at *p* < 0.05. All statistical analyses were performed in R [52].

## 5. Conclusions

Our study addressed the effect of lowland forest fragment size on plant species richness and diversity with a focus on ecologically important groups (native, alien, alien invasive, and ancient forest indicator species). Our results revealed that the effects of fragmentation are complex and differ among studied plant groups. By decreasing forest fragment size, overall plant richness, and the richness of native and ancient forest indicators, plants per plot, and their diversity, decrease. On the other hand, the effects of forest fragment size on alien plants and, among them, invasive ones, were more complex. Their richness, proportion, and cover declined in forest fragments larger than 23 ha to the value recorded for the unfragmented forest. Our results confirm the high conservation value of unfragmented habitats because the highest proportion of AFI was listed there. However, the presence of alien plants in the same plots indicates that even large and diverse forest ecosystems are susceptible to biological invasions. According to our results, we suggest sustaining forest fragments of at least 13 ha in the studied agricultural landscape to enable their species conservation role in the future. Cumulative plant species richness was higher in smaller forest fragments than in unfragmented forest Murska šuma. Even though small forest fragments have high importance for biodiversity in otherwise biologically depleted landscapes, higher nature conservation value in terms of native plant richness, diversity, and compositional quality should be attributed to large unfragmented forest habitats. Our results serve as a case study that can help determine critical sizes of temperate lowland forest fragments which should be maintained in a landscape if we want to preserve their nature conservation value.

## Figures and Tables

**Table 1:** List of alien plant species and ancient forest indicator plants (AFI) present in forest fragments of Apaško polje and Dravsko polje (N = 47) and unfragmented forest Murska šuma (N = 29). Invasive alien plants (according to Jogan et al. [28], *P. americana* was later recognized as invasive) are in bold.

Alien Plant	% in Forest Fragments	% in Unfragmented Forest	AFI	% in Forest Fragments	% in Unfragmented Forest
***Acer negundo* L.**	2.13	3.45	*Acer campestre* L.	17.02	100
***Ailanthus altissima* (Mill.) Swingle**	4.26	0	*Asarum europaeum* L.	12.77	37.93
*Allium* sp.	2.13	0	*Athyrium filix-femina* (L.) Roth	0	37.93
***Ambrosia artemisiifolia* L.**	0	3.45	*Berberis vulgaris* L.	2.13	0
***Duchesnea indica* (Andrews) Focke**	4.26	0	*Brachypodium sylvaticum* (Huds.) Beauv.	19.15	51.72
***Erigeron annuus* (L.) Pers.**	4.26	6.9	*Carex pallescens* L.	2.13	0
*Fraxinus americana* L.	0.00	6.9	*Carex pendula* Huds.	0	44.83
***Impatiens glandulifera* Royle**	8.51	20.69	*Carex remota* L.	2.13	44.83
***Impatiens parviflora* DC.**	36.17	3.44	*Carex sylvatica* Huds.	12.77	89.66
*Juglans nigra* L.	0	13.79	*Circaea lutetiana* L.	4.26	86.21
*Oxalis fontana* Bunge	4.26	0	*Clematis vitalba* L.	2.13	0
***Parthenocissus quinquefolia* (L.) Planch.**	6.38	0	*Convallaria majalis* L.	2.13	0
***Phytolacca americana* L.**	17.02	0	*Cornus mas* L.	0	3.45
*Populus x canadensis* Moench	0	6.9	*Cornus sanguinea* L.	19.15	65.52
*Prunus laurocerasus* L.	4.26	0	*Corylus avellana* L.	19.15	24.14
*Prunus serotine* Ehrh.	12.77	0	*Crataegus* sp.	14.89	44.83
*Quercus rubra* L.	2.13	0	*Dactylis glomerata subsp. lobata* (Drejer) Lindb. F.	2.13	0
***Robinia pseudoacacia* L.**	34.04	0	*Dentaria bulbifera* L.	0	17.24
***Rudbeckia laciniata* L.**	4.26	0	*Dryopteris carthusiana* (Vill.) H.P. Fuchs	14.89	0
***Solidago gigantea* Aiton**	25.53	34.48	*Dryopteris filix-mas* (L.) Schott	4.26	6.90
*Taxus baccata* L.	2.13	0	*Euonymus europaeus* L.	31.91	31.03
			*Festuca gigantea* (L.) Vill.	2.13	0
			*Festuca heterophylla* Lam.	6.38	0
			*Galeobdolon montanum* (Pers.) Rchb.	14.89	89.66
			*Luzula luzuloides* (Lam.) Dandy and Wilmott	12.77	0
			*Luzula pilosa* (L.) Willd.	4.26	0
			*Maianthemum bifolium* (L.) F.W.Schmidt	0	3.45
			*Melampyrum* sp.	2.13	0
			*Milium effusum* L.	0	44.83
			*Oxalis acetosella* L.	6.38	31.03
			*Paris quadrifolia* L.	0	58.62
			*Polygonatum multiflorum* (L.) All.	2.13	34.48
			*Pteridium aquilinum* (L.) Kuhn	17.02	0
			*Pulmonaria officinalis* L.	6.38	100
			*Ranunculus auricomus* L.	2.13	0
			*Rhamnus catharticus* L.	4.26	0
			*Sanicula europaea* L.	0	6.90
			*Scrophularia nodosa* L.	2.13	10.34
			*Stachys sylvatica* L.	0	79.31
			*Stellaria holostea* L.	0	6.90
			*Stellaria nemorum* L.	0	13.79
			*Tilia cordata* Mill.	19.15	0
			*Tilia platyphyllos* Scop.	2.13	0
			*Ulmus laevis* Pall.	0	79.31
			*Ulmus minor* Mill.	14.89	0
			*Vaccinium myrtillus* L.	8.51	0
			*Viburnum opulus* L.	14.89	10.34
			*Vinca minor* L.	2.13	0
			*Viola reichenbachiana* Jord. ex Boreau	4.26	55.17

**Table 2:** Summary of linear (y = a *q + r), quadratic (y = a *q + b *q^2^ + r), and polynomial (y = a *q + b *q^2^ + c *q^3^ + r) regression analyses of forest fragment size effect on plant species richness, composition, and diversity of plant communities. Significant coefficient estimates a, b, and c are in bold and marked as ** <0.01, * <0.05, ^+^ <0.1. Grey color represents statistically non-significant models. Murska šuma is not included in the analysis.

	Adjusted R^2^	F-Statistic	Coefficient Estimates			Standard Error
			a	b	c	
**Plant Species Richness**						
Overall	0.07	**2.63 ^+^**	10.66 ^+^	6.52		5.45
Native	0.05	2.21	6.34	8.02		4.87
Alien	0.19	4.58 **	4.32 **	−1.50	−2.65 ^+^	1.43
IAS	0.03	1.43	1.73	−0.67	−1.53	1.16
AFI	0.08	3.09 ^+^	1.27	5.43 *		2.24
% Native	0.17	5.79 **	−22.87 **	15.79 ^+^		8.17
% Alien	0.20	4.79 **	22.87 **	−15.79 ^+^	−12.52	8.04
% IAS	0.03	1.50	9.14	−7.91	−6.44	6.47
% AFI	0.19	6.45 **	−13.80	38.92 **		11.49
**Composition**						
Alien/Native	0.19	4.63 **	0.30 *	−0.22 ^+^	−0.20 ^+^	0.11
IAS/Native	0.05	1.87	0.13	−0.11	−0.11	0.09
AFI/Other native	0.31	8.02 **	−0.24	0.95 **	−0.67 **	0.24
**Diversity**						
Shannon	0.13	7.64 **	1.24 **			0.45
Simpson’s	0.13	7.95 **	0.40 **			0.14
Evenness	0.09	5.67 *	0.31 *			0.13

**Table 3:** Species richness, proportion, and cover of native, alien, invasive alien, and ancient forest indicator plants in the unfragmented forest, large, medium, and small forest fragments. Average values with standard deviation are given. Different uppercase and lowercase letters represent significant differences at *p* < 0.05 for Tukey HSD or Mann–Whitney pairwise test, respectively.

Species Richness	Native		Alien		IAS		AFI	
	Average	SD	Average	SD	Average	SD	Average	SD
Unfragmented	27.90 ^A^	5.27	1.24 ^a^	1.41	0.72 ^a^	0.92	12.38 ^A^	2.31
Large	16.88 ^B^	4.12	3.00 ^b^	1.85	1.75 ^a^	1.49	4.50 ^B^	2.73
Medium	11.91 ^B^	4.29	1.61 ^a,b^	1.53	0.83 ^a^	1.15	2.52 ^B^	1.75
Small	14.25 ^B^	5.57	0.81 ^a^	0.98	0.75 ^a^	0.93	3.88 ^B^	2.60
**Proportion (%)**								
Unfragmented	96.99 ^a^	3.43	3.76 ^a^	4.04	2.10 ^a^	2.60	43.03 ^a^	5.99
Large	84.12 ^b^	11.23	15.88 ^b^	11.23	9.15 ^a^	8.20	21.68 ^b^	13.66
Medium	88.95 ^b^	8.52	11.05 ^b^	8.52	5.69 ^a^	6.76	17.55 ^b^	9.65
Small	95.58 ^a^	5.37	4.42 ^a^	5.37	4.03 ^a^	4.96	25.71 ^b^	15.34
**Cover (%)**								
Unfragmented	93.15 ^a^	5.40	4.23 ^a^	8.27	1.89 ^a^	2.86	59.37 ^A^	17.02
Large	65.84 ^b^	15.91	11.95 ^b^	7.94	7.34 ^a^	8.15	18.88 ^B^	19.37
Medium	51.00 ^b^	21.33	5.74 ^a^	13.70	2.38 ^a^	4.46	8.33 ^B^	15.01
Small	55.49 ^b^	18.78	6.36 ^a,c^	17.03	5.88 ^a^	15.86	16.41 ^B^	24.43

**Table 4:** Diversity indices in an unfragmented forest, large, medium, and small forest fragments. Different uppercase and lowercase letters represent significant differences at *p* < 0.05 for Tukey HSD or Mann–Whitney pairwise test, respectively.

	Species Richness		Shannon		Simpson’s		Evenness	
	Average	SD	Average	SD	Average	SD	Average	SD
Unfragmented	29.14 ^A^	6.15	2.54 ^a^	0.26	0.87 ^a^	0.03	0.76 ^a^	0.04
Large	19.88 ^B^	3.59	1.99 ^b^	0.29	0.8 ^b^	0.06	0.67 ^b^	0.07
Medium	13.52 ^C^	5.06	1.47 ^c^	0.32	0.68 ^c^	0.11	0.58 ^b^	0.12
Small	15.06 ^B,C^	6.1	1.5 ^b,c^	0.63	0.65 ^c,b^	0.21	0.55 ^b^	0.17

**Table 5:** Classification of forest vegetation based on three species’ characteristics: origin, invasiveness, and ancient forest indicator plants *sensu* Hermy et al. [14].

Classification	Expected Forest Fragment Size Effect	Expected Effect of Forest Cover in Buffer
Origin (native vs. alien) plant species	Higher alien plant invasion potential in smaller fragments	The higher richness of alien plant species at lower forest cover
Invasive alien species (IAS)	Higher richness and proportion of IAS in smaller fragments	Higher IAS richness with decreasing forest cover
Ancient forest indicator plant species (AFI)	Higher richness and proportion in unfragmented forest	The increasing richness and proportion with increasing forest cover

## Data Availability

The datasets generated during and/or analyzed during the current study are available from the corresponding author upon reasonable request.
